# The Multifaceted Role of miR-211 in Health and Disease

**DOI:** 10.3390/biom15081109

**Published:** 2025-08-01

**Authors:** Juan Rayo Parra, Zachary Grand, Gabriel Gonzalez, Ranjan Perera, Dipendra Pandeya, Tracey Weiler, Prem Chapagain

**Affiliations:** 1 Rutgers New Jersey Medical School, Rutgers University, 185 South Orange Avenue, Newark, NJ 07103, USA; jdr278@njms.rutgers.edu; 2 Herbert Wertheim College of Medicine, Florida International University, 11200 SW 8th St AHC2, Miami, FL 33199, USA; zgran007@med.fiu.edu (Z.G.); ggonz288@med.fiu.edu (G.G.); tweiler@fiu.edu (T.W.); 3 Department of Neurosurgery, Oncology, Sidney Kimmel Comprehensive Cancer Center, School of Medicine, Johns Hopkins University, 5505 Hopkins Bayview Cir, Baltimore, MD 21224, USA; jperera2@jh.edu; 4 Johns Hopkins All Children’s Hospital, 501 6th Ave S, St. Petersburg, FL 33701, USA; 5 Department of Physics, Biomolecular Sciences Institute, Florida International University, 11200 SW 8th St, Miami, FL 33199, USA

**Keywords:** microRNA-211, microRNA, gene regulation, cancer biomarkers, cellular metabolism, TGF-β signaling, post-transcriptional regulation, therapeutic targets, miR-211

## Abstract

MicroRNA-211 (miR-211) is a versatile regulatory molecule that plays critical roles in cellular homeostasis and disease progression through the post-transcriptional regulation of gene expression. This review comprehensively examines miR-211’s multifaceted functions across various biological systems, highlighting its context-dependent activity as both a tumor suppressor and oncogene. In physiological contexts, miR-211 regulates cell cycle progression, metabolism, and differentiation through the modulation of key signaling pathways, including TGF-β/SMAD and PI3K/AKT. miR-211 participates in retinal development, bone physiology, and protection against renal ischemia–reperfusion injury. In pathological conditions, miR-211 expression is altered in various diseases, particularly cancer, where it may be a useful diagnostic and prognostic biomarker. Its stability in serum and differential expression in various cancer types make it a promising candidate for non-invasive diagnostics. The review also explores miR-211’s therapeutic potential, discussing both challenges and opportunities in developing miRNA-based treatments. Understanding miR-211’s complex regulatory interactions and context-dependent functions is crucial for advancing its clinical applications for diagnosis, prognosis, and targeted therapy in multiple diseases.

## 1. Introduction

Micro-RNAs (miRNAs) represent a class of small non-coding RNAs, approximately 22 nucleotides in length, that play critical roles in regulating gene expression at the post-transcriptional level. These small RNAs regulate gene expression by repressing translation or inducing the degradation of messenger RNAs (mRNAs) across a wide range of species.

miRNAs were first discovered in 1993 during experiments on the nematode, *Caenorhabditis elegans* [1]. In these experiments, Lee et al. discovered a heterochronic switch gene in the worm that functioned via complementary binding to mRNA and inhibition of its translation [2]. Heterochronic switch genes are a group of genes that control the timing of the development of an organism, delaying the precocious onset of subsequent stages of development by inhibiting or inducing the expression of specific genes. For example, the lin-4 gene, a small non-coding strand of RNA, was found to have antisense complementarity to lin-14 mRNA [2]. The lin-4:lin-14 interaction temporarily downregulates lin-14 during postembryonic development, with important implications in several developmental milestones in *C. elegans*. At the time, this method of post-translational gene suppression was thought to be exclusive to *C. elegans*. However, in the early 2000s, let-7, another heterochronic switch gene that produces a 21-nucleotide RNA sequence complementary to many genes essential for adult development from the late larval stage, was discovered [3]. Homologs of the let-7 gene were subsequently found in humans [4].

Although these initial discoveries involved miRNAs within the heterochronic gene pathway, subsequent research revealed miRNAs involved in a wide variety of important cellular processes including apoptosis, proliferation, and metabolic regulation, amongst others [5]. miRNAs exert their regulatory functions primarily by binding complementary sequences on 3′ untranslated regions (UTRs) of their target RNA transcripts [5]. Typically, miRNAs negatively regulate their targets, depending on how the microRNA base pairing influences the secondary structure of the target mRNA [5]. This regulation can lead to mRNA degradation and subsequently translational regulation.

miRNAs have also been found to have a positive regulatory influence on gene expression [6]. One mechanism occurs via disruption of the activity of repressive miRNAs. Additionally, miRNAs can indirectly influence mRNA translation through binding to regulatory factors and recruiting them to express specific genes [7]. A recent study revealed a role for miRNAs in upregulating the α-2,6-sialyltransferases *ST6GAL1* and *ST6GAL2*, enzymes that control terminal modification of α-2,6-sialic acid [7]. Sialic acids are known to play a major role as signaling molecules in certain diseases and can drive cancer development [8]. Therefore, dysregulation of these miRNAs could have significant phenotypic consequences.

The biologic synthesis of miRNAs begins with transcription in the nucleus by RNA polymerase II [9]. miRNA genes are typically clustered within introns of other protein-coding genes and are regulated by their own regulatory sequences [9,10]. Initially, a long transcript containing a hairpin-like structure that houses several miRNA transcripts is produced [10,11]. This structure is known as pri-miRNA. The pri-miRNA is then processed by Drosha, a nuclear RNAse III that, along with DGCR8, also known as Pasha, forms the pri-miRNA processing complex [11,12]. The pri-miRNA processing complex is responsible for cleavage of pri-miRNA to release another miRNA precursor segment, approximately 70 nucleotides in length, termed pre-miRNA [12]. Pre-miRNA is produced in the nucleus and exported via the protein exportin 5 (EXP5) in a RAN-GTP-dependent process [13]. Once in the cytoplasm, the pre-miRNA transcript is cleaved by DICER, a type III RNA endonuclease, generating a double-stranded miRNA duplex ~22 nucleotides in length [14]. This miRNA duplex is associated with an Argonaut (AGO) protein as part of the RNA-induced silencing complex (RISC) [15].

**Figure 1:**
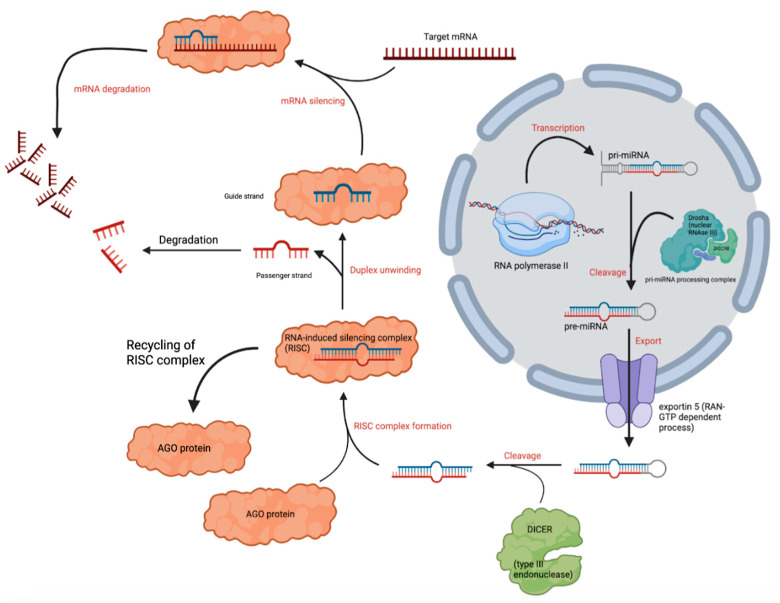
miRNA biogenesis and action on target mRNA; image created with BioRender.com.

Humans produce four closely related AGO proteins (AGO1 to AGO4), each of which can bind miRNA, but only AGO2 has been shown to have the “slicer” activity responsible for direct cleavage of target mRNAs [16]. Target recognition by the RISC complex occurs via recognition of a 2-7 nucleotide sequence at the 5′ end of the miRNA termed the seed sequence. This makes AGO2 a critical player in miRNA pathways, facilitating target mRNA degradation or translational repression depending on the complementarity of the target sequence [17]. While AGO2 is unique in its endonuclease activity, the other AGO proteins (AGO1, AGO3, and AGO4) also participate in gene silencing through translational repression [16,17]. After association with the RNA-induced silencing complex (RISC), the miRNA duplex unwinds, producing a single-stranded active miRNA guide strand and releasing its complement passenger strand for degradation. This unwinding step and the broader biogenesis pathway are illustrated in Figure 1.

Mature miRNAs arise from either the 5′ or 3′ arm of the hairpin precursor and are annotated accordingly as -5p or -3p. The -5p form often represents the functionally dominant strand, such as miR-211-5p, which is the most widely studied and biologically active variant of miR-211 in most contexts. miR-204, a closely related paralog of miR-211, shares a similar seed sequence and is functionally redundant in some systems, especially within the retina and joint tissues. Its contribution alongside miR-211 warrants parallel discussion in developmental and disease contexts.

## 2. Biogenesis of miRNA Variants

Unproductive miRNAs are formed because of defects or inefficiencies at any point from transcription to RISC incorporation and stabilization, resulting in molecules that are either degraded or fail to regulate gene expression [18,19]. Other primary sequences in various pri-miRNAs may serve as important recognition features that further guide microprocessor cleavage [20].

Improper recognition and cleavage of the pri-miRNA produces an alternative pre-miRNA with 5′ ends that can vary in length from canonical pre-miRNA [20]. Since DICER cleavage occurs at a fixed distance from the 5′ end, this non-canonical pre-miRNA produces variant miRNAs, or isomiRs [21]. The resulting isomiRs may have different seed sequences and thus target and regulate different mRNAs. For example, the miRNA-9 isomiR (miR-9-alt) has 539 targets that differ from the canonical miRNA-9 (miR-9-can), which may have functional and clinical consequences. In the context of low-grade gliomas, miR-9-alt was shown to downregulate *BACE2*, *COL1A2*, and *FGL2*, all of which are associated with increased survival in patients [22]. Further research is needed to establish if these mechanistic consequences of isomiR expression change outcomes for patients.

Malfunctions in various steps of the miRNA biogenesis pathway can lead to isomiR generation, and the development of high-throughput and next-generation sequencing has allowed for their identification and classification [21,23]. IsomiRs arise due to post-transcriptional modification of the miRNA transcript, resulting in one of five possible classifications: canonical miRNAs, 5′ isomiRs, 3′ isomiRs, polymorphic isomiRs, and mixed type isomiRs. Canonical miRNAs arise from the DICER and DROSHA pathway and are found in miRNA databases. 5′ and 3′ isomiRs vary in nucleotide length at the 5′ and 3′ ends, respectively [21]. Polymorphic isomiRs are the same length as their canonical miRNA counterparts, but vary in sequence, while mixed-type isomiRs show changes in both nucleotide length and sequence [21]. Alterations in the seed sequence can lead to variations in target recognition and thus variations in the functional outcome of miRNA silencing.

In recent years, there have been significant advances in understanding the connections between various diseases and specific miRNAs. Among these, miR-211 has emerged as a significant regulator of diverse biological processes including proliferation, cellular differentiation, and apoptosis. Its dysregulation has been implicated not only in cancer but also in other pathologies such as neurological disorders, cardiovascular diseases, and inflammatory conditions. miR-211 functions as a regulatory molecule with roles that can vary depending on the disease context, acting either as a protective factor or as a contributor to disease progression.

Here we review the multifaceted role of miR-211 in normal physiology and disease development and progression, exploring the underlying molecular mechanisms and its potential as a therapeutic target. By deepening our understanding of the role of miR-211 in various diseases, we can advance our strategies for diagnosis, prognosis, and treatment across various pathological conditions.

## 3. Role of miR-211 in Normal Human Biology and Physiology

### 3.1. miR-211 Regulation of TGF-β Signaling and Cell Cycle Control

miR-211 plays a multifaceted role in regulating the cell cycle and maintaining tissue homeostasis by targeting key components of cell cycle machinery and signaling pathways [24]. It inhibits G0/G1 phase progression by downregulating genes such as cyclin D1 and cyclin-dependent kinase (CDK) inhibitors, thereby limiting uncontrolled cell proliferation across multiple cell types, including melanocytes, neural crest-derived cells, osteoblasts, chondrocytes, and hepatocytes [25,26,27].

Beyond cell cycle arrest, miR-211 is integral to cellular differentiation. In neural crest cells, for example, it fine-tunes the expression of developmental transcription factors crucial to melanocyte lineage specification [28]. In retinal, hepatic, and skeletal tissues, miR-211 also supports proper differentiation and function [29,30,31,32].

Several of miR-211’s direct targets—KCNMA1, IGF2R, and TGFBR2—govern membrane excitability, growth factor signaling, and cell fate decisions (Table 1). Its regulation of these targets underpins homeostatic balance across neuronal, hepatic, and epithelial systems [33,34,35].

**Figure 2:**
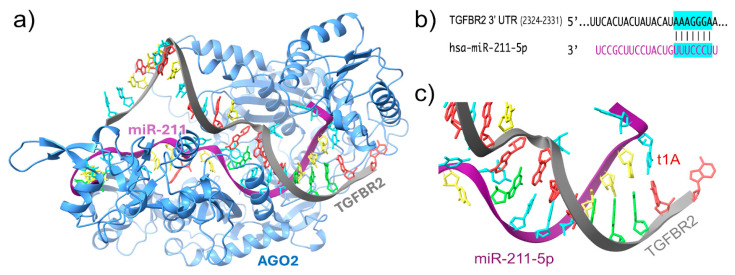
Structural and sequence-level depiction of miR-211 targeting TGFBR2. (**a**) AlphaFold 3.0-predicted model of the Ago2-miR-211-TGFBR2 complex. (**b**) Seed region pairing between miR-211 and the 3′ UTR of TGFBR2 (nucleotides 2324–2331), predicted via TargetScan. (**c**) Magnified view showing canonical Watson–Crick base pairing at the miRNA–mRNA interface.

A key mechanism of action is miR-211’s repression of TGFBR2, a type II receptor in the transforming growth factor-beta (TGF-β) signaling pathway, which is widely expressed in epithelial cells, fibroblasts, endothelial cells, and immune cells [36]. miR-211 binds to the 3′ untranslated region (UTR) of TGFBR2 mRNA, leading to its degradation and translational repression [37]. This interaction acts as a molecular brake, ensuring that TGF-β signaling remains within a physiological range (Figure 2).

In normal tissue, ligand binding to TGFBR2 enables the phosphorylation of the type I receptor (TGFBR1), which in turn phosphorylates SMAD2/3 [38]. These R-SMADs complex with SMAD4 and translocate to the nucleus to regulate gene transcription [33,34,35]. When properly modulated, this cascade promotes quiescence, differentiation, and controlled immune responses. miR-211 maintains signaling equilibrium by keeping TGFBR2 levels in check, thus preventing pathological outcomes such as unchecked proliferation, fibrosis, or immune evasion [38,39].

For example, SMAD3—a downstream effector of TGF-β—is a known mediator of immunosuppression. By constraining TGFBR2 expression, miR-211 reduces excessive SMAD3 activation, preserving immune function in T cells and other leukocyte populations [40]. Similarly, TGF-β signaling induces p15 and p21, CDK inhibitors that suppress cell cycle progression [41]. miR-211’s modulation of TGFBR2 helps ensure these signals do not excessively suppress regenerative capacity [42,43].

Thus, in healthy states, miR-211 serves as a homeostatic modulator: restraining TGF-β pathway activity to promote tissue integrity, regulate immune balance, and prevent maladaptive cellular remodeling. Its dysregulation can lead to aberrant fibrotic or proliferative responses, as discussed in Section 4.1 and Section 4.4.

### 3.2. miR-211 Targets in Chromatin Regulation and PI3K/AKT Signaling

Beyond its role in TGF-β modulation, miR-211 also regulates transcriptional dynamics and growth factor signaling through a diverse network of downstream targets. These processes, biological contexts, and functional consequences are highlighted in Table 2.

One notable target is structure-specific recognition protein 1 (Ssrp1), a component of the Facilitates chromatin transcription (FACT) complex. Ssrp1 interacts with histones H2A and H2B to promote nucleosome disassembly, enabling transcriptional elongation [44]. In epithelial progenitor cells, Ssrp1 also acts as a co-activator of the transcription factor p63, a member of the p53 family essential for epithelial development and tissue regeneration [44,45]. Dysregulation of Ssrp1 contributes to pathological proliferation, especially in hepatocellular carcinoma, inflammation, and autoimmune disease, highlighting the importance of miR-211’s post-transcriptional control of this factor [46,47].

miR-211 also plays a crucial role in modulating the PI3K/AKT pathway, which governs cellular metabolism, survival, and proliferation [48]. This pathway is frequently hyperactivated in malignancy and autoimmunity. By restraining PI3K/AKT signaling under physiological conditions, miR-211 helps maintain cellular quiescence and prevents excessive growth signals [48,49]. Interestingly, miR-211 itself is a transcriptional target of bone morphogenetic protein 2 (BMP2)—a member of the TGF-β superfamily—further integrating it into homeostatic growth control networks [50].

miR-211 regulates several transcription factors and signaling proteins that orchestrate cellular differentiation and metabolic homeostasis: SOX11 and SOX4, which drive progenitor cell fate decisions, are repressed by miR-211 to prevent aberrant or premature differentiation [50]. SPARC, SNAI1, and ZEB2, key mediators of epithelial-to-mesenchymal transition (EMT), are modulated to maintain epithelial integrity and limit pro-metastatic transitions [24,51]. ACSL4, an enzyme involved in fatty acid metabolism and ferroptosis sensitivity, is regulated by miR-211 to prevent lipid peroxidation and metabolic stress [52].

At the transcriptional level, miR-211 expression is regulated by transcription factor-12 (TCF12), a helix-loop-helix transcription factor that binds to E-box motifs (CANNTG) in the promoter regions of target genes [53]. TCF12 is prominently expressed in developing tissues such as skeletal muscle, neural tissue, immune cells, and bone, aligning with sites of active miR-211 expression and function [54,55]. Through this regulatory axis, miR-211 integrates developmental and metabolic signals to support tissue differentiation, regeneration, and structural homeostasis.

Table 1 summarizes validated and predicted targets of miR-211 across diverse tissues, reinforcing its context-dependent regulatory roles in normal physiology. To reduce redundancy across sections, we have consolidated the major signaling pathways regulated by miR-211 across diverse contexts in Table 2.

**Table 1:** The various genes and proteins targeted by microRNA-211 (miR-211), outlining their roles in cellular differentiation and homeostasis. Data compiled from publicly available transcriptomic atlases including GTEx, Human Protein Atlas, and tissue-specific RNA-seq studies. Relative abundance assessed based on consensus expression across >3 studies.

Gene	Pathological Interaction with miR-211	Normal Function	Tissue Expression	Primary Cell Types	Ref.
KCNMA1	Upregulated; promotes cancer progression	Regulates membrane potential and Ca^2+^ signaling	Brain, smooth muscle, endocrine tissues	Neurons, smooth muscle cells, adrenal gland cells	[33]
IGF2R	Upregulated; enhances tumor growth	Mediates uptake of IGF-2	Liver, kidney, muscle	Hepatocytes, renal tubules, myocytes	[34]
TGFBR2	Increased expression contributes to metastasis	Receptor in TGF-β pathway; regulates growth and differentiation	Lung, liver, heart, immune cells	Alveolar cells, hepatocytes, cardiomyocytes, T cells	[35]
TCF12	Upregulated; promotes cancer progression	Transcriptional regulation of development	Embryonic and adult tissues	Stem cells, differentiating cells	[53,54,55]
SOX11	Elevated in tumors	Neurogenesis and differentiation	Developing nervous system	Neural progenitors, differentiating neurons	[50]
SOX4	Upregulated; contributes to metastasis	Cell fate determination	Bone marrow, lymphoid tissue	HSCs, lymphocytes, mesenchymal cells	[50]
SPARC	Promotes cancer migration	Cell–matrix remodeling	Bone, skin, connective tissues	Osteoblasts, fibroblasts, stromal cells	[51]
SNAI1	Induces EMT	EMT regulation	Embryonic and tumor tissues	Epithelial cells, cancer stem cells	[24]
ZEB2	Upregulated; drives EMT and metastasis	EMT and neural crest development	Neural crest, epithelia	Neural crest cells, epithelial cells	[24]
ACSL4	Elevated expression affects lipid metabolism in cancer	Long-chain fatty acid activation	Liver, brain, adipose tissue	Hepatocytes, neurons, adipocytes	[52]
SSRP1	Promotes chromatin remodeling in tumor cells	Chromatin regulation via FACT complex	Proliferating tissues	Tumor cells, chromatin-regulating cells	[44,45,46,47]
Runx2	Upregulated; contributes to osteosarcoma	Osteoblast differentiation and bone formation	Bone, cartilage	Osteoblasts, chondrocytes	[31,56,57,58]

**Table 2:** Summary of major signaling pathways regulated by miR-211 in health and disease. The table lists key pathways, their validated or predicted miR-211 targets, biological contexts in which these regulatory interactions occur, and the functional consequences associated with their modulation. Functional outcomes vary based on cellular environment and disease state. References correspond to specific studies supporting each interaction. This table consolidates mechanistic themes discussed across Section 2, Section 3 and Section 4 to reduce narrative redundancy and enhance interpretability.

Signaling Pathway	Validated or Predicted Target(s)	Biological Context	Functional Consequence	Reference(s)
TGF-β/SMAD	TGFBR2	Melanocytes, renal epithelium, T cells	Represses TGF-β signaling, limits SMAD2/3 activation, prevents fibrosis and apoptosis	[35,38,40]
PI3K/AKT	PI3K-associated factors (e.g., via SSRP1)	Synoviocytes, chondrocytes, epithelial cells	Reduces AKT activation; controls inflammation and abnormal proliferation	[48,49]
Cell Cycle Regulation	Cyclin D1, CDK6, CDC25B	Cancer cells, synoviocytes	Induces G0/G1 arrest; inhibits proliferation and promotes cell cycle checkpoint activation	[24,25,26,27]
Chromatin Remodeling	SSRP1	RA synoviocytes, epithelial progenitors	Inhibits FACT complex; restrains NF-κB and p53 pathway activation	[44,59]
EMT/Metastasis	SNAI1, ZEB2, SPARC	Cancer (e.g., cervical, renal, oral)	Inhibits EMT, reduces cell migration and metastatic potential	[24,51]
Metabolic Regulation	ACSL4, pyruvate metabolism enzymes	Melanoma, retina	Regulates oxidative metabolism; protects against metabolic stress and ferroptosis	[52,60,61]
BMP2/TGF-β Superfamily	BMP2	Pancreatic cancer	Suppresses tumor growth and invasion	[50]
STAT3 (indirect)	Indirect via SSRP1 and PI3K/AKT	Synovial fibroblasts (RA)	Reduces inflammatory cytokines; restores apoptosis sensitivity	[59]

### 3.3. miR-211 and Bone Physiology

The paralogs miR-204 and miR-211 are essential for the maintenance of joint health and have been implicated in the prevention of osteoarthritis (OA). In vitro studies demonstrate that both miRNAs regulate Runx2 [31], a critical transcription factor involved in the development and maintenance of bone, cartilage, and teeth [56,57]. Runx2 is required for osteoblast differentiation and activates downstream pathways, such as fibroblast growth factor receptor (FGFR) signaling, to sustain cartilage homeostasis [56]. Additionally, Runx2 governs the fate of chondrocytes, determining whether they remain transient or differentiate into permanent cartilage [58].

Mesenchymal progenitor cells (MPCs) deficient in miR-204 and miR-211 exhibit excessive Runx2 accumulation, increased MPC proliferation, abnormal bone formation, and in vivo development of OA-like features [31]. Moreover, such MPCs also accumulate cartilage-degrading enzymes, matrix metalloproteinase 13 (MMP13) and a disintegrin and metalloproteinase with thrombospondin motifs 5 (ADAMTS5), which further promote joint degeneration [62].

Aberrant expression of Ssrp1, a known target of miR-211, has also been linked to rheumatoid arthritis (RA) [59]. In this context, decreased miR-211 levels lead to elevated Ssrp1, overactivation of Nuclear factor kappa beta (NF-κB) signaling, suppression of the p53 pathway, and enhanced synovial cell proliferation [58]. In vivo studies show that miR-211 downregulation of Ssrp1 can mitigate RA symptoms by reducing pathological cell growth and inflammation as well as temporomandibular joint osteoarthritis [59,63].

To extend these findings in vivo, a double-knockout (dKO) mouse model was developed, deleting both miR-204 and miR-211 in multiple joint tissues. These dKO mice exhibited widespread OA-like symptoms throughout the joint, not limited to cartilage alone [63]. Pathological features included severe synovial hyperplasia, osteophyte formation, subchondral sclerosis, and upregulation of nerve growth factor (NGF). Increased NGF expression in MPCs may promote aberrant nerve ingrowth and contribute to OA-associated pain [63].

Interestingly, fewer dKO pups were born than expected, suggesting either prenatal lethality or interference from the Prx1-Cre transgenic strain, which shares chromosomal proximity with miR-211 on mouse chromosome 7 [64]. Although the dKO mice appeared phenotypically normal in early life, they developed progressive joint degeneration with age, making this model particularly relevant to the human OA disease course [64]. The extensive joint pathology observed in these mice underscores the joint-wide regulatory role of miR-204 and miR-211, highlighting their importance beyond cartilage-specific functions.

### 3.4. miR-211 and Human Eye Development

Recent studies have revealed critical roles for miR-211 in regulating retinal metabolism, photoreceptor maintenance, and cell survival [29]. Its paralog, miR-204, has been implicated in insulin regulation and glucose homeostasis, with dysregulation contributing to complications such as diabetic retinopathy. Additionally, point mutations in miR-204 have been linked to inherited retinal dystrophies, underscoring the need to investigate miR-211’s function in ocular physiology [65].

Transcriptome analyses demonstrate that miR-211 regulates gene expression networks controlling glucose, pyruvate, and lipid metabolism in retinal cells [60]. In vitro studies support its role as a metabolic switch, helping retinal cells adapt to fluctuating oxygen and nutrient conditions [60]. miR-211-deficient melanoma cells exhibit abnormal energy metabolism, and similar metabolic dysregulation likely occurs in miR-211-deficient retinal cells [61]. These effects position miR-211 as a key modulator of metabolic homeostasis, especially under environmental stress.

Furthermore, oxidative stress and mitochondrial dysfunction resulting from miR-211 loss are increasingly implicated in retinal degeneration [66]. miR-211 deficiency may impair lipid homeostasis, promoting degeneration through metabolic failure rather than apoptosis. In this context, miR-211 acts as a protective factor against degenerative stress in the retina.

In vitro studies show that the inactivation of miR-211 in mice leads to progressive cone dysfunction followed by cone cell loss, while rod photoreceptors remain largely unaffected. Notably, outer nuclear layer (ONL) thickness is preserved even in older mice, and rod bipolar cell (RBC) function remains intact at seven months, indicating selective vulnerability of cone cells [29,60,65]. By 18 months, approximately 50% of cones are lost.

Though miR-204 may offer partial compensation, context-dependent differences in targeting suggest distinct regulatory roles for the two paralogs. For example, CHOP (C/EBP homologous protein), a hallmark of ER stress-mediated apoptosis, remains unchanged in miR-211 knockout mice, as do TUNEL staining results, indicating a lack of classical apoptosis [67,68]. These findings suggest that cone loss proceeds via non-apoptotic pathways, such as ferroptosis, necroptosis, or metabolic collapse—a novel avenue for further investigation.

## 4. Role of miR-211 in Disease Pathology

### 4.1. miR-211’s Role in Renal Hypoxia/Reoxygenation and Ischemia/Reperfusion Injury

miR-211 appears to protect the kidneys from injury caused by hypoxia/reoxygenation (H/R) and ischemia/reperfusion (I/R) [69]. Overexpression of miR-211 protected renal cells from H/R injury and accelerated recovery from I/R injury in mice. Low miR-211 levels due to H/R or I/R injury increased cell death and resulted in more severe kidney damage, while increasing miR-211 levels helped to reverse these harmful effects [69].

As discussed previously, Smads are intracellular proteins phosphorylated by TGFβR intracellular domains, causing them to dissociate and translocate to the nucleus, where they act as transcription factors. Smads act as tumor suppressors by inhibiting cell proliferation and promoting apoptosis, mainly by inducing p15 and p21, which are known CDK inhibitors [39,41]. When miR-211 levels are low, TGFβR2 transcription increased, thus inactivating the TGF-β/SMAD pathway and inducing cell death. Additionally, the study discovered that H/R treatment reduced p-SMAD3 expression in renal epithelial NK-2 cells, and this reduction was worsened by inhibiting miR-211 or overexpressing TGFβR2. Conversely, knockdown of TGFβR2 or using miR-211 analogs increased p-SMAD2/3 levels, highlighting the importance of the miR-211/TGFβR2/TGF-β/SMAD3 axis in apoptosis during H/R injury.

In vivo experiments supported these findings, showing that reducing miR-211 expression in mice with renal I/R injury increased cell death and worsened kidney function [69]. Conversely, increasing miR-211 protected against cell death, reduced kidney damage, and preserved kidney function. The study concluded that miR-211 protects against kidney I/R injury by regulating TGFβR2/TGF-β/SMAD3 signaling [67], suggesting that targeting miR-211 could be a promising therapeutic approach for renal I/R injury.

### 4.2. miR-211’s Role in Atherosclerosis and Vascular Calcification

Contributing to its diverse functions, there have been recent studies of the clinical relevance of serum miR-211-5p in vascular calcification (VC) in patients with end-stage renal disease (ESRD). VC is a fundamental pathological process in cardiovascular diseases in patients with ESRD. Vascular smooth muscle calcification is largely driven by high serum phosphorus levels, which downregulates miR-211, subsequently causing the accumulation of Runx2, which promotes calcification [70].

The relative expression of serum miR-211-5p was assessed in patients who had vascular calcifications by qRT-PCR. Results showed that serum miR-211-5p levels were significantly reduced in the calcified group and progressively decreased with VC severity. The calcified group showed significantly elevated levels of creatinine and were more likely to have hypertension than the control group [70]. Additionally, calcium-phosphate product, fetuin-A, and renal creatinine clearance were substantially lower in patients with vascular calcifications than in the control group. Serum miR-211-5p had a high diagnostic accuracy for identifying VC progression in ESRD patients. Additionally, Kaplan–Meier and Cox regression analyses suggested that miR-211-5p could be an independent prognostic biomarker for ESRD patients. Therefore, miR-211-5p could be a potential diagnostic and prognostic marker for VC in patients with ESRD.

In another study, the expression of miR-211-5p was assessed in patients with atherosclerosis (AS). Kaplan–Meier curve and Cox regression analysis were used to determine the prognostic significance of miR-211-5p transcript levels in patients with AS. The results indicated that miR-211-5p levels were significantly lower in the sera of asymptomatic AS patients than in healthy control groups [71]. Patients with asymptomatic AS were divided into high and low expression groups based on the average serum expression of miR-211-5p. Over five years, twenty-five AS patients experienced cardiovascular events, including eight strokes, three myocardial infarcts, and fourteen transient ischemic attacks, of whom 19 were from the low miR-211-5p expression group and six were from the high expression group [69]. Patients with lower miR-211-5p expression had shorter event-free survival than those with higher expression levels [71]. Additionally, multivariable Cox regression analysis suggested that miR-211-5p could be an independent prognostic factor for cardiovascular events in AS patients. These findings note that expression levels of miR-211-5p could independently predict a patient’s likelihood of having a cardiovascular event, regardless of other factors.

### 4.3. miR-211 Immunological Disease

Rheumatoid arthritis (RA) is a chronic, multifactorial autoimmune condition influenced by genetic and environmental factors. RA is characterized by inflammation, leading to abnormal synovial hyperplasia and joint destruction. Wang et al. explored the impact of miR-204/211 on RA, focusing on synovial inflammation and proliferation. miRNA-204 is downregulated in RA synovial tissues and regulates RA fibroblast-like synoviocyte (FLS) survival via STAT3 [59]. Consistently, miR-204 and miR-211 levels were significantly reduced in collagen-induced arthritis (CIA) mouse FLS. In vitro experiments showed that overexpression of miR-204/211 alleviated synovial inflammation, inhibited migration, and promoted apoptosis of CIA FLS, while knockdown had the opposite effect. Overexpression of miR-204 and miR-211 decreased proinflammatory cytokines and increased anti-inflammatory cytokines, whereas their knockdown increased inflammation. miR-204 and miR-211 were found to attenuate inflammation by regulating NF-κB p65 translocation [59].

Additionally, miR-204 and miR-211 influenced cell proliferation by regulating cell cycle proteins, blocking the G0/G1 phase, and altering the expression of Ccnd1 and CDK inhibitors. The study also demonstrated that miR-204 and miR-211 inhibited aberrant proliferation by modulating PI3K/AKT signaling [59].

**Figure 3:**
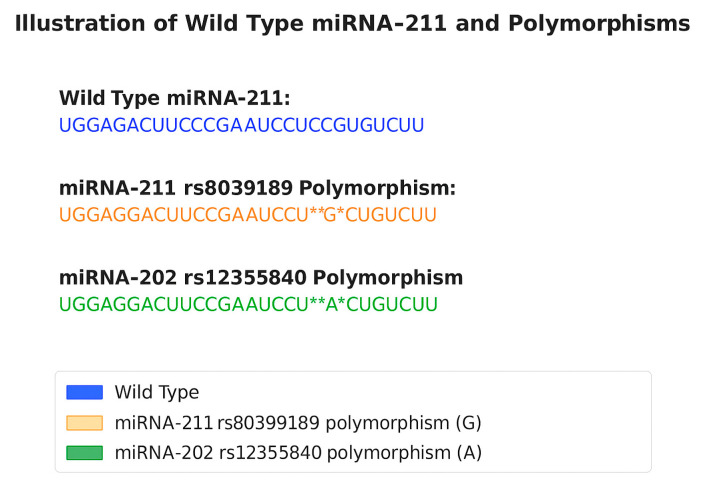
Wild-type miR-211 and vitiligo polymorphisms [56]. Illustration of wild-type miRNA-211 and polymorphic variants. The wild-type miRNA-211 sequence is shown in blue. The rs8039189 polymorphism in miRNA-211 is highlighted in orange and involves a G substitution (G) at a specific position. The rs12355840 polymorphism in miRNA-202 is shown in green, with an A substitution (A) at the corresponding position. Colored asterisks indicate the location of single-nucleotide polymorphisms (SNPs). Sequences are aligned to highlight shared structure. This figure illustrates how minor base changes can alter miRNA integrity and potentially impact function.

There have also been recent attempts to assess the relationship between 3 miRNA polymorphisms and the risk of vitiligo. Figure 3 shows the wild-type variant and polymorphism of miR-211 that are relevant to vitiligo. Vitiligo is an autoimmune disease characterized by the immune cell mediated deterioration of melanocytes. The miR-211 rs8039189 polymorphism may protect against the development of vitiligo, while the miR-202 rs12355840 polymorphism may increase susceptibility to the condition. There was no significant relationship between the miR-1238 rs12973308 polymorphism and susceptibility to vitiligo [72].

### 4.4. miR-211 in Cancer

miR-211’s bifunctional role as an oncogene or tumor suppressor is influenced by its tissue-specific expression, alternative splicing (5p vs. 3p), interaction with distinct cofactors, and tumor microenvironment. For instance, the presence of co-regulators like Microphthalmia-associated transcription factor (MITF) in melanocytes vs. TCF12 in hematopoietic cells may underlie the divergence in downstream targets [73]. Further, the abundance of competing endogenous RNAs (ceRNAs) and isomiR variation can modulate target accessibility, altering its phenotypic outcome.

The dysregulation of and pathologic variants in miR-211 can contribute to disease progression. Aberrant upregulation of miR-211 expression can strongly suppress TGF-β receptor expression, reducing cellular sensitivity to TGF-β-mediated growth inhibition [74]. This loss of regulatory control can contribute to unchecked cellular proliferation and tumor progression, particularly in cancers where TGF-β signaling initially acts as a tumor suppressor [75]. Conversely, if miR-211 expression is suppressed, excessive TGF-β signaling may promote fibrosis, immune evasion, and metastasis, as seen in certain advanced malignancies [74,75,76,77,78,79,80]. The interplay between miR-211 and the TGF-β pathway highlights its critical role in maintaining cellular homeostasis and underscores its potential as a therapeutic target in oncology and fibrosis-related disorders.

We have seen that miR-211 can be implicated as both a tumor suppressor or an oncogene, depending on the cellular and disease context. As a tumor suppressor, miR-211 can regulate genes involved in cell proliferation and apoptosis, preventing tumor growth. For instance, in certain cancers such as breast cancer, miR-211 suppresses tumor cell growth, migration, and invasion by downregulating targets like CDC25B, which are crucial for cell cycle progression [81]. However, miR-211 can also act as an oncogene. In melanoma, it contributes to cancer progression by promoting cellular invasion and resistance to targeted therapies, like *BRAF* V600E inhibitors, through its interaction with signaling pathways such as ERK5 [82]. This pleiotropism highlights miR-211’s complex role in regulating cancer-related pathways, where it can either inhibit or promote tumor growth based on the specific molecular environment.

As expression of miR-211 is associated with tumor metastasis and aggression, it may have considerable prognostic value. Several studies have shown expression levels of miR-211 are lower in cancers than in normal tissue, implicating it as a potential tumor suppressor, including in bladder cancer, renal cancer, hepatocellular carcinoma, epithelial ovarian cancer, oral squamous cell carcinoma, gastric cancer, and melanoma [25,82,83,84,85,86,87,88,89]. In melanoma, miR-211 has been shown to significantly influence metabolic pathways. Loss of miR-211 altered energy metabolism, particularly through dysregulation of pyruvate and lipid metabolism [82,87]. This metabolic shift could enable melanoma cells to survive under hypoxic conditions by adapting their bioenergetic processes. Such alterations are associated with increased oxidative stress, which can further contribute to cancer progression and resistance to therapy.

Other studies have demonstrated an oncogenic role for miR-211 due to overexpression in certain cancers [90]. Studies exploring miR-211 as a prognostic indicator in gastric cancer revealed overexpression in gastric cancer tissue compared with healthy mucosa [89]. This expression was also prognostic, as a relatively high miR-211 expression correlated with a higher incidence of lymph node metastasis. Subsequent studies confirmed the finding, with gastric cancer patients exhibiting a significantly higher plasma miR-211-5p levels and a more accurate diagnosis when serum measurements of miRNA-195-5p were integrated into the screening process [89]. Increased miR-211 levels have also been observed in oral carcinoma, and ectopic expression of miR-211 caused growth of oral squamous cell carcinoma cells [88].

miR-211 is shown to enhance carboplatin-induced DNA damage by interfering with DDR genes. Ovarian cancer cells treated with carboplatin showed significantly more DNA damage when transfected with miR-211 [91]. miR-211 also plays a role in the pathogenesis of T cell lymphoblastic lymphoma. miR-211 overexpression inhibited the growth of T cell lymphoblastic leukemia, slowing the rate of DNA synthesis [46,53]. Furthermore, in vivo observations showed that high levels of miR-211 decreased tumor mass and volume in mice. It is worth noting that this study used a xenograft tumor model and, furthermore, one major target of miR-211 was found to be an mRNA transcript of TCF12, a protein that acts as a transcription factor recognizing the CANNTG sequence [53]. The results indicated that transfection of miR-211, contrary to the miR-211 inhibitor, resulted in more significant DNA damage in ovarian cancer cells treated with carboplatin.

Further studies have shown how miR-211 regulates TGF-β-related molecules such as bone morphogenetic protein 2 (BMP2), for example in the context of miR-211-5p expression and pancreatic cancer severity. In vitro experiments showed that increased expression of miR-211-5p decreases proliferation and metastatic potential of pancreatic cancer cells [50]. Furthermore, BMP2 was identified as a direct target of miR-211-5p. BMP2 is derived from the TGF- β class [50]. Their experiments further concluded that BMP2 directly drives the growth and migration of pancreatic cancer cells and, in vivo, upregulation of miR-211-5p reduces tumor development. Mouse models with transfected cells containing miR-211-5p mimics showed significantly reduced tumor volume and mass. Additionally, Li et al. demonstrated that increased expression of miR-211-5p was associated with increased survival time. This study suggests that the relationship between miR-211-5p and BMP2 can be used as a prognostic marker for patients with pancreatic cancer.

Certain cancers have a particularly poor prognosis, often attributed to their late diagnosis and resistance to standard therapeutics. Current diagnostic techniques for cancer often involve the invasive procedure of tissue biopsy of suspected cancerous tissues, with associated drawbacks of prolonged recovery times, risk of complications, and significant emotional and physical stress. There is therefore growing interest in less invasive diagnostic and screening tools, including serum genetic testing. New tumor markers are desperately needed to improve patient outcomes by securing an earlier diagnosis.

However, in cancer, dysregulation of miR-211 leads to an imbalance in these regulatory pathways. When miR-211 expression is downregulated, oncogenes such as KCNMA1, IGF2R, and TGFBR2 are upregulated, contributing to uncontrolled proliferation and enhanced survival of cancer cells [92]. In melanoma, loss of miR-211 increases KCNMA1 expression, promoting metastasis [92]. Similarly, overexpression of IGF2R enhances invasive potential, while elevated TGFBR2 levels facilitate immune evasion and fibrosis, contributing to tumor progression [79,92].

miR-211’s role in tumorigenesis is highly context-dependent and can vary significantly depending on the cancer type. Although the mechanism underlying this variability has not been extensively described, circulating miR-211 can modulate many genes simultaneously. This dual oncogenic-tumor suppressive role is influenced by factors such as the specific target gene, other regulatory molecules, or environmental factors.

Standardizing expression profiles of miR-211 across different cancer subtypes is the next step in developing effective screening and diagnostic applications. This would involve first establishing reference ranges through large-scale population studies in healthy patients. Due to the large-scale variability in miRNA expression profiles for each cancer subtype, with some upregulating and others downregulating miR-211, each will need its own reference range.

The resilience and stability of biomarkers are significant factors to consider when discussing their potential in clinical diagnostics. The relative stability of miR-211 in plasma suggests potential as a biomarker. Unlike larger RNA molecules, miRNAs circulate in a stable form resistant to degradation by endogenous RNase activity [93]. In a comparative study, miRNAs extracted from lung carcinoma demonstrated significantly greater stability to RNase A than large molecular weight RNAs [94]. Another study highlighted the remarkable resilience of miRNAs to acidic conditions found in human breast milk, where they remained stable even when treated in an acidic solution for 1 h [95]. Additionally, the concentrations of miRNAs in frozen plasma samples have been shown to remain constant from 5 to 14 years after freezing [93].

qRT-PCR is often used to quantify miRNAs in biological samples, often in conjunction with sequencing to confirm that miRNA expression profiles are indeed miRNA products and not other forms of small RNA or degraded RNA fragments [96,97]. In the early 2000s, novel techniques were developed to screen mature serum miRNAs using stem-loop RT-PCR with high sensitivity, specificity, and precision [97]. In this process, the transcription primer has a stem-loop structure, allowing for accurate discrimination between precursor and mature forms of miRNA. These stem loop primers have been shown to better discriminate miRNAs than traditional RT-PCR primers and to accurately quantify small RNA molecules in various biological samples, including tissue and plasma. This method enables profiling of miRNA expression from as little as 10 pg of total RNA [96].

Given these attributes, miR-211 is a promising future biomarker for screening and monitoring cancer progression.

## 5. Future Directions and Limitations

### 5.1. miR-211: Therapeutic Potential and Translational Challenges

The exploration of microRNAs (miRNAs) as therapeutic targets has introduced a new frontier in cancer treatment. miR-211, in particular, functions as a tumor suppressor in multiple malignancies, including medulloblastoma (MB) and melanoma, through modulation of metabolic and transcriptional programs [98,99]. In MB, miR-211 is significantly downregulated across subgroups (SHH, Group 3, Group 4), and its reintroduction inhibits proliferation, induces apoptosis, and reduces invasiveness [98]. Similarly, in melanoma, restoration of miR-211 expression attenuates metastatic potential by targeting genes involved in oxidative stress response and cellular invasion [99].

Therapeutic strategies involving miRNAs fall into two major approaches: (i) inhibition of oncogenic miRNAs using antisense oligonucleotides (ASOs), locked nucleic acid (LNA) inhibitors, or miRNA sponges [100]; and (ii) restoration of tumor-suppressive miRNAs via synthetic mimics. For tumors with miR-211 downregulation, synthetic miRNA mimics have shown preclinical efficacy in suppressing tumor growth [101]. Delivery platforms such as lipid nanoparticles (LNPs), viral vectors (e.g., AAV), and engineered exosomes are under active investigation to enhance tumor-specific targeting and in vivo stability [102].

Despite these advances, multiple translational barriers remain. Circulating miRNAs are rapidly degraded, necessitating protective delivery systems [102,103]. Moreover, miRNAs inherently target multiple mRNAs, raising concerns regarding off-target effects and systemic toxicity [103]. Clinical trials of other miRNA-based therapies, such as MRX34 (miR-34a mimic), have been halted due to immune-related adverse events, highlighting the need for optimized delivery vehicles and thorough immune compatibility profiling [104]. Currently, no miR-211-targeted therapies are in clinical trials, though preclinical findings suggest promising directions.

Importantly, due to the pleiotropic nature of miRNA regulation, combinatorial approaches—such as miR-211 mimics with immunotherapy or chemotherapy—may synergistically enhance treatment efficacy and overcome drug resistance mechanisms [99]. Rigorous pharmacodynamic studies and detailed biodistribution analyses will be essential for advancing these approaches into clinical use.

### 5.2. Dual Roles of miR-211 in Cancer: Context-Dependent Mechanisms

While miR-211 frequently acts as a tumor suppressor, reports of oncogenic behavior have emerged, particularly in glioma and certain breast cancers [105]. The dualistic role of miR-211 likely reflects context-dependent molecular determinants. (i) Cell-Type-Specific Cofactors: Transcriptional regulators such as MITF modulate miR-211 expression and downstream target engagement, influencing whether miR-211 enforces differentiation or promotes survival [73]. (ii) Competing Endogenous RNAs (ceRNAs): The abundance of lncRNAs and pseudogenes that sequester miR-211 can redirect its targeting behavior, shifting its role from suppressive to permissive [106]. (iii) Chromatin and Epigenetic Landscape: The accessibility of target sites is influenced by the local epigenetic context, which may dictate functional output in a tissue-specific manner [107]. These complexities underscore the need for integrative studies combining transcriptomics, epigenomics, and single-cell analyses to dissect the determinants of miR-211 function in distinct cellular environments.

### 5.3. Extracellular Vesicle-Based Delivery of miR-211 and Other Therapeutic miRNAs

Recent advances in extracellular vesicle (EV) research have introduced a promising modality for the delivery of miRNA-based therapeutics, including miR-211. EVs such as exosomes are naturally secreted membrane-bound particles capable of transferring bioactive molecules—including proteins, lipids, and RNA—between cells [108]. Their biocompatibility, ability to traverse biological barriers, and natural role in intercellular communication make them attractive vehicles for RNA-based therapies.

Multiple studies have demonstrated that EVs can be engineered to carry synthetic or endogenous miRNAs for therapeutic purposes [109,110]. A recent study reviewed recent strategies for engineering EVs to deliver therapeutic miRNAs directly to tumor sites [111]. These methods include surface modification with targeting ligands, endogenous expression of miRNAs in donor cells, and exogenous loading via electroporation or sonication [111]. Their review highlighted successful applications of miRNA-loaded EVs in targeting cancers such as hepatocellular carcinoma and glioblastoma, where miRNA therapy reduced tumor growth and altered tumor microenvironments [112].

In a related study, Pottash et al. evaluated the anti-inflammatory potential of combinatorially loaded EVs containing miR-146a, miR-155, and miR-223 [113]. Using sonication to co-load multiple miRNAs into HEK293T-derived EVs, they demonstrated significant IL-6 suppression in LPS-stimulated macrophages, with promising effects observed in a murine endotoxemia model. These findings suggest the feasibility of using multi-miRNA-loaded EVs for immunomodulation and inflammation control in cancer therapy.

Complementing these results, Doyle and Wang offered a broad review of EV therapeutics, emphasizing the clinical translational potential of miRNA-based delivery systems [114,115]. They discussed the advantages of EVs over synthetic nanoparticles, including lower immunogenicity and better cellular uptake, while also addressing current challenges such as standardization, large-scale production, and regulatory considerations.

Together, these findings underscore the potential of EV-based systems for delivering miR-211 in oncologic contexts, particularly in melanoma and medulloblastoma where its tumor-suppressive functions are well established. Future studies should explore miR-211-EV delivery platforms tailored to specific tumor microenvironments and investigate combination regimens with chemotherapy or immunotherapy. Such approaches may overcome limitations of systemic delivery and enable precise reprogramming of oncogenic pathways.

### 5.4. Gaps in Knowledge of miR-211 Biology

While miR-211 inactivation mainly affects cone photoreceptor cells, this miRNA is not highly expressed in this cell type. This may be due to cones’ high susceptibility to retinal metabolism/catabolism alterations. The miR-211 paralog miRNA-204 also plays a key role in photoreceptor function and maintenance despite low expression in these cells. These observations support the idea that miRNAs can have significant roles even in cell types where they are expressed at low levels. However, cone dysfunction in miR-211^−/−^ mice might also be due to non-cell autonomous effects. The mode of action of miR-211, its relevance in controlling retinal metabolism and catabolism processes, and its function in other retinal cell types like bipolar cells require further study.

In osteoarthritis (OA), recent studies utilizing miR-204/211 double knockout (dKO) mice have revealed severe joint degeneration beyond cartilage damage, implicating these miRNAs in joint-wide homeostasis. The observation of synovial hyperplasia, increased NGF expression, and aberrant Runx2 accumulation in dKO mice suggests that miR-211 modulates mesenchymal progenitor cell (MPC) differentiation and inflammatory signaling. Additionally, osteophyte formation resembling endochondral ossification suggests an active developmental program driven by miR-211 loss. The observed reduction in dKO pup viability may also indicate prenatal lethality, necessitating embryonic studies to assess broader developmental roles.

Moreover, Li et al. demonstrated that miR-211-5p directly suppresses BMP2 in pancreatic cancer, attenuating proliferation and migration. These findings warrant deeper investigation into miR-211-5p’s downstream networks and potential feedback loops in the TGF-β superfamily.

Taken together, the multifaceted nature of miR-211 underscores both its translational promise and the importance of detailed mechanistic studies to fully realize its clinical potential.

## 6. Conclusions

MicroRNA-211 is a versatile regulator of multiple biological systems and disease contexts. miR-211 can function as both a tumor suppressor and an oncogene, with its exact role depending on specific cellular and molecular context. It contributes to key physiological processes, including cell cycle regulation, differentiation, and metabolism, and has critical implications in diseases such as cancer, rheumatoid arthritis, and kidney injury. The mechanisms through which miR-211 exerts its effects—primarily through modulation of signaling pathways like TGF-β/SMAD and PI3K/AKT—suggest it could act as a therapeutic target.

miR-211’s differential expression in various cancers highlights its potential as a diagnostic and prognostic biomarker, offering avenues for non-invasive cancer screening. Furthermore, miR-211’s stability in serum enhances its suitability as a biomarker. Beyond cancer, the regulatory influence of miR-211 in inflammatory and metabolic processes, as well as its protective effects in renal and cardiovascular conditions, underscores its wide therapeutic relevance. However, the context-dependent functionality of miR-211, acting as both an oncogene and tumor suppressor, presents a unique challenge for therapeutic intervention. This duality necessitates an approach that carefully considers cellular context to effectively harness miR-211’s therapeutic potential.

Future research should focus on unraveling these complexities and establishing standardized expression profiles for miR-211 across various diseases. Additionally, exploring the development of specific miR-211 isomiRs could provide a novel mechanism for selectively targeting distinct cellular contexts. Efforts should also be directed toward manipulating miR-211 gene expression to ensure precise temporal and spatial delivery, thereby maximizing therapeutic efficacy. These advancements will be essential to harness miR-211’s full potential in diagnostic and therapeutic applications, paving the way for innovative treatments.

## Data Availability

No new data were created or analyzed in this study.
